# Real-world data of Azvudine-induced hepatotoxicity among hospitalized COVID-19 patients in China: a retrospective case-control study

**DOI:** 10.3389/fphar.2025.1558054

**Published:** 2025-06-04

**Authors:** Yuanguo Xiong, Hao Xin, Cai Shi, Xianxi Guo, Ying Chen, Caifei Huang, Fuwang Ma, Ge Yang, Jian Yang

**Affiliations:** ^1^ Department of Pharmacy, Renmin Hospital of Wuhan University, Wuhan, China; ^2^ Department of Pharmacy, Qingdao Third People’s Hospital Affiliated to Qingdao University, Qingdao, China; ^3^ Department of Pharmacy, First Affiliated Hospital of Army Medical University, Chongqing, China

**Keywords:** COVID-19, Azvudine, hepatotoxicity, risk factors, real-world data

## Abstract

**Background:**

The COVID-19 pandemic, caused by the SARS-CoV-2 virus, has led to global health crisis. Although several antiviral drugs have been used to mitigate the severity and mortality of COVID-19, the safety profile remained a critical concern. Azvudine, a new nucleoside analog, has been approved for emergency use in China for COVID-19. However, the incidence and risk factors associated with Azvudine-induced hepatotoxicity in hospitalized patients remained unclear.

**Objects:**

To assess the prevalence, risk factors, clinical patterns, and outcomes of Azvudine-induced hepatotoxicity by real-world data.

**Methods:**

We conducted a single-center retrospective case-control study at Renmin Hospital of Wuhan University, including patients administered Azvudine for COVID-19 treatment between December 2022 and May 2023. Univariate and multivariate logistic regression analyses were preformed to assess risk factors for Azvudine-associated or -induced hepatotoxicity. Receiver operating characteristic (ROC) curve analysis was performed to calculate the area under the ROC curve (AUC).

**Results:**

In total, 669 patients were included in the Azvudine-associated hepatotoxicity research. 47.1% patients exhibited hepatotoxicity, abnormal liver function on admission [OR: 5.55 (3.94–7.90), *P <* 0.001] and antithrombotic drugs [OR: 1.79 (1.27–2.54), *P =* 0.001] were independent predictors of Azvudine-associated hepatotoxicity, with the area under the ROC curve (AUC) was 0.756 [95% CI: 0.719–0.792, *P <* 0.001]. Further studies of Azvudine-induced hepatotoxicity revealed 294 cases, of which 27.2% showed hepatotoxicity. The concomitant use of antivirals [OR: 3.80 (1.47–10.1), *P =* 0.006] and anticoagulant drugs [OR: 3.12 (1.77–5.61), *P <* 0.001], particularly Ganciclovir [OR: 4.11 (1.45–12.2), *P =* 0.008], Low-Molecular-Weight Heparin Calcium [OR: 3.00 (1.69–5.33), *P <* 0.001], and Enoxaparin [OR: 2.68 (0.99–7.10), *P =* 0.047], were significantly associated with an increased risk of hepatotoxicity. Most hepatotoxicity cases were mild, and recovered or improved after drug withdrawal and treatment, whereas severe cases contributed to the progression of the primary disease and increased mortality risk.

**Conclusion:**

Our study provided evidence of the significant association between Azvudine and hepatotoxicity in hospitalized COVID-19 patients. These findings underscored the importance of monitoring liver function during Azvudine treatment and caution against concomitant use of certain medications. Further research was warranted to elucidate the mechanisms underlying Azvudine-induced hepatotoxicity and optimize clinical management strategies.

## 1 Introduction

The COVID-19 pandemic, triggered by the SARS-CoV-2 virus, has led to millions of fatalities worldwide, with enduring repercussions on global health systems, economic stability and social structures (Kevadiya et al., 2021; Kwok, 2022; Baden et al., 2021; Rubin, 2021). As of 2024, the COVID-19 pandemic remains a persistents public health challenge, characterized by the emergence of novel variants and sustained international efforts in disease mitigation (Chow et al., 2023; Nanaw J, 2024). To solve the problem, several antiviral drugs, such as Remdesivir, Molnupiravir, and Nirmatrelvir-Ritonavir, have been utilized in the treatment of COVID-19 following to WTO guidelines (Han et al., 2024a; Grein et al., 2020). Additionally, Azvudine (2′-deoxy-2′-β-fluoro-4′-azidocytidine), a novel nucleoside analog, has gained emergency approval for the Omicron variant surge in China (General Office of the National Health Commission, 2022; Deng et al., 2023).

Azvudine is a pro-drug that undergoes intracellular phosphorylation by deoxycytidine kinase phosphorylated in the cytoplasm to form its active metabolite. The active compound exerts antiviral effects primarily through inhibition of the viral RNA-dependent RNA polymerase (RdRp) of viruses (Zhang et al., 2021; Yu and Chang, 2020). Initially developed for HIV treatment, Azvudine has demonstrated favorable efficacy and tolerability profiles in Chinese patients during 48-week therapeutic regiments. Recent studies have revealed its potent activity against SARS-CoV-2 virus with dual mechanisms: effective suppression of viral RNA replication and immunodulatory effects through thymus-mediated lymphocyte profile enhancement (Ren et al., 2020; Yang et al., 2024a). The pharmacological properities make Azudine a promising therapeutic candidate for COVID-19. Clinical trials have demonstrated Azvudine significant antiviral efficacy against COVID-19. Prior studies have revealed comparable clinical outcomes between Azvudine and Nirmatrelvir-Ritonavir in hospitalized patients, with no statistically significant differences observed in 28 days all-cause mortality, composite disease progression, and clinical improvement (Han et al., 2024b; Shang et al., 2024). The findings provide robust evidence supporting Azvudine effectiveness as an alternative treatment for COVID-19, with safety and efficacy profile. Compared to Nirmatrelvir-Ritonavir, Azvudine demonstrated comparable clinical efficacy while showing a significantly lower risks of composite disease progression in non-severe COVID-19 patients, indicated Azvudine might offer superior clinical benefits over Nirmatrelvir-Ritonavir in certain patient populations (Yu and Chang, 2022; Wang S. et al., 2024). Further supporting evidence from a retrospective case-control study by Su et al. demonstrated comparable therapeutic efficacy between Azvudine and Nirmatrelvir-Ritonavir, with no statistically significant differences observed in key clinical outcomes including recovery time, mortality rates, and hospitalization duration (Su et al., 2024). Meta-analysis further indicated that Azvudine may be preferable to Nirmatrelvir-Ritonavir for elderly patients (aged over 75 years old), primarily due to its significantly lower risk of drugs interaction while maintaining comparable efficacy (Wang Y. et al., 2024; Shang et al., 2024). Furthermore, pharmacoeconomic analyses demonstrate that Azvudine exhibited a siginicantly lower incremental cost-effectiveness ratio (ICER) compare to Nirmatrelvir-Ritonavir (Yang et al., 2024b). The enhanced cost-efficiency profile improved treatment accessibitity, particularly in resource-limited and lower-income populations. In summary, current clinical and pharmacoeconomic evidence positions Azvudine as a clinically effective and cost-efficient therapeutic option for COVID-19 management, particularly valuable for resource-constrained healthcare systems.

Previous research has demonstrated that SARS-CoV-2 infection not only affects the respiratory system but also involves multiple organ dysfunction (MODS), thus inevitably leading to the increase in all-cause mortality (Zhong et al., 2020; Alqahtani and Schattenberg, 2020). Notably, hepatic dysfunction occurs in up to half of all reported cases (Alqahtani and Schattenberg, 2020; Allison et al., 2023). Medications have been recognized as one of the contributing factor to hepatocellular injure in COVID-19 infections (Zhong et al., 2020; Wen-Shu Hu, 2022; Rodriguez-Espada et al., 2024). Notably, antiviral drugs such as Remdesivir, and Nirmatrelvir-Ritonavir have been associated with an elevated risk of drug-induced hepatotoxicity (Allison et al., 2023; Xie et al., 2020; Naseralallah et al., 2022). The safety and tolerability of Azvudine as a novel nucleoside analog has remain critical considerations, particularly in patients with pre-exsiting liver impairment. According to the manufacturer’s label, Azvudine may cause mild-to-moderate liver dysfunction in isolated instances, with approximately 35% of patients exhibiting modest elevation in liver enzyme. Clinical studies have reported hepatotoxicity ranges from 13.5% to 38.4%, udine among COVID-19 patients receiving Azvudine (Lan et al., 2024; Zhi et al., 2023; He et al., 2023; Liu et al., 2024; Yang et al., 2023; Liu S. et al., 2023). However, the generalizability of these findings remains uncertain due to limited sample size. Consequently, further large-scale studies are warranted to clarify the safety profile of Azvudine in COVID-19 treatment.

This study utilized real world data to evaluate the prevalence, risk factors, clinical manifestations, and outcomes of Azvudine-associated hepatotoxicity among in hospitalized COVID-19 patients. The findings aim to establish a comprehensive safety profile of Azvudine and provide evidence-based guidance for clinical decision-making in COVID-19 management.

## 2 Methods

### 2.1 Study design and ethics approval

This single-center retrospective case-control study was conducted at the Renmin Hospital of Wuhan University, a tertiary medical center with approximately 7300 beds in China. This study protocol was approved by the Ethics Committee of the Renmin Hospital of Wuhan University (Approval No.: WDRY2024-K003), with waiver of informed consent granted due to the retrospective study.

### 2.2 Patient inclusion

The diagnosis of COVID-19 has made by infectious disease and internal medicine clinicians based on clinical signs, a chest computed tomography scan, and a positive COVID-19 nucleic acid test result. Patients with respiratory rates≥30 breaths/minute, SpO_2_≤93% on room air, or PaO_2_/FiO_2_ ≤ 300 mmHg were categorized as severe cases, while other cases were classified as non-severe (General Office of the National Health Commission, 2022). Patients who were Azvudine for COVID-19 treatment between December 2022 to May 2023 were included. Exclusion criteria for Azvudine associated hepatotoxicity based on the following criteria: 1) Negative COVID-19 nucleic acid test results, 2) Incomplete laboratory data (lack of baseline liver function data within 7 days prior to treatment initiation or absence of follow up, and 3) Azvudine treatment less than 3 days (Shi et al., 2021; Zhao et al., 2024; Lu et al., 2024). Additional exclusion criteria for Azvudine-induced hepatotoxicity included: 1) pre-existing liver disease and 2) abnormal liver function tests at admission.

**FIGURE 1:**
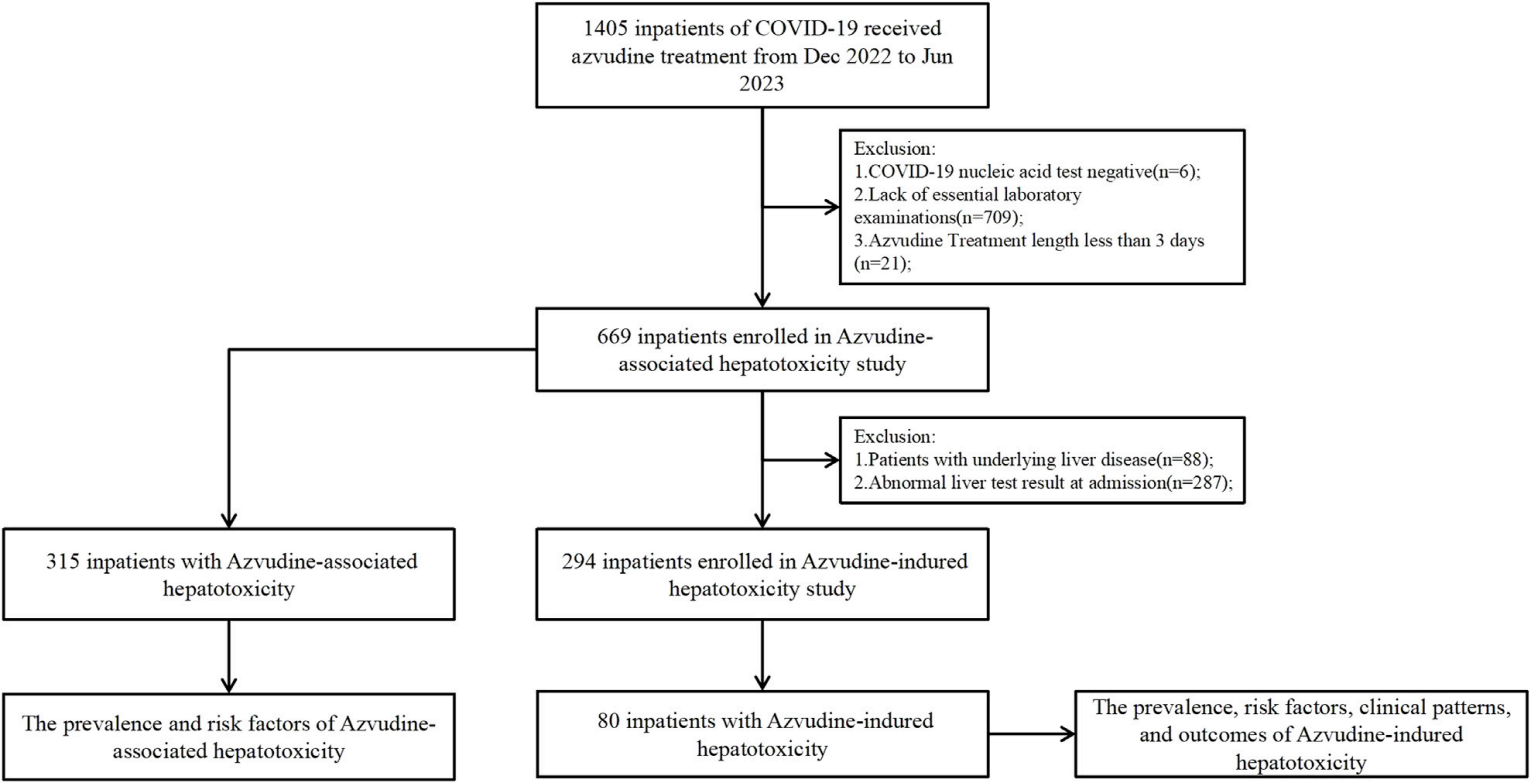
Identification of Azvudine-associated or -induced hepatotoxicity among COVID-19 hospitalized patients during the study period.

The patients’ comprehensive data including: demographic characteristics, COVID-19 severity, Azvudine dosage, duration of treatment liver disease history, serial liver function tests, comorbidities, and concomitant medications. A schematic flowchart delineating the methodology for identifying Azvudine-associated and Azvudine-induced hepatotoxicity cases was shown in Figure 1.

### 2.3 Evaluation of hepatotoxicity

Hepatotoxicity was defined as liver function biochemical abnormalities s when meet at least one of the following biochemical criteria: 1) alanine aminotransferase (ALT) > 40U/L; 2) aspartate transaminase (AST) > 35U/L; 3) alkaline phosphatase (ALP) > 135U/L; 4) gamma–glutamyltransferase (GGT) > 45U/L; 5) total bilirubin (TB) > 23 μmol/L (Yu et al., 2017). All values represent the upper limit of normal (ULN) for our laboratory reference ranges. In this study, patients who developed hepatotoxicity after Azvudine treatment were categorized into the Abnormal group, while those without hepatotoxicity were classified as the Normal group.

The causality of Azvudine-induced hepatotoxicity was evaluated using the updated Roussel Uclaf Causality Assessment Method (RUCAM) (Danan and Teschke, 2016; Zhao et al., 2021), with cases scoring below 3 on the RUCAM scale being excluded from analysis due to insufficient evidence for establishing causal relationship between liver injury and Azvudine administration. To classify the clinical type of Azvudine-induced hepatotoxicity, the R value was calculated using the following formula: R = [ ALT/ (ALT ULN)]/ [ALP/ (ALP ULN)]. Based on established criteria (Andrade et al., 2019a; Andrade et al., 2019b), drug-induced liver injury was categorized into three clinical types: 1) hepatocellular injury (R ≥ 5); 2) cholestatic injury (R ≤ 2); 3) mixed injury (2 < R < 5).

The severity of Azvudine-induced hepatotoxicity was classfied according to the biological criteria Common Terminology Criteria for Adverse Events (CTCAE) Version 5.0, based on the ULN thresholds of ALT, ALP, and GGT as follows: 1) Grade 1 (Mild): elevation of ALT or ALT ≥1 × ULN and <3 × ULN, elevation of ALP or GGT ≥1 × ULN and <2.5 × ULN; 2) Grade 2 (Moderate): elevation of ALT or ALT ≥3 × ULN and <5 × ULN and elevation of ALP or GGT ≥2.5 × ULN and <5 × ULN; 3) Grades 3 and 4 (severe induced liver injury, DILI): elevation of ALT or ALT ≥5 × ULN and elevation of ALP or GGT ≥5 × ULN.

### 2.4 Prognosis of azvudine-induced hepatotoxicity

The prognosis of Azvudine-induced hepatotoxicity was categorized based on post-treatment liver function records was as follows: 1) Complete Recovery: liver enzyme levels returned to within normal limits (below ULN) or baseline values; 2) Partial Improvement: ALT/AST decreased to below 3 × ULN or ALP/GGT decreased to below 2.5 × ULN; 3) Aggravation: liver test values exceeded previous peak levels; 4) Unknown: Insufficient follow-up data for proper assessment.

### 2.5 Statistical analysis

Statistical analysis was conducted using SPSS Statistics version 26.0 (IBM Corp., Armonk, NY, USA). Continuous variables with normal variables were reported as mean ± standard deviation (SD), whereas non-normally distributed variables were presented as medians (interquartile range, IQR). Independent sample t-tests and Mann-Whitney U tests were for comparisons. Categorical variables were analyszed using either chi-square test or fisher’s exact test, as appropriate. For multivariable analysis, variables demonstrating a potential association (*P* < 0.20) in univariate analysis were entered into a logistic regression modelIndependent risk factors for Azvudine-associated and Azudine-induced hepatoxicity were identified through stepwise logistic regression. A two-tailed *P* value < 0.05 was considered statistically significant for all analysis.

We performed sensitivity analyses to evaluate the robustness and generalizability of primary findings, following established methodological approaches (Hykin et al., 2019; Liu ZQ. et al., 2023). In the current analysis, we mainly conducted sensitivity analyses on two specific subgroups. Age-stratified analysis: We introduced an age-treatment interaction term in the regression model to specifically examine potential effect modification in patients aged more than 35 years old. Allergy adjusted analysis: we systematically exclude patients with documented drug allergy histories and re-analyzed the primary outcomes to assess the potential confounding effect of medication hypersensitivity. These rigorous sensitivity assessments were designed to:

1) Verify the consistency of treatment effects across clinically relevant subgroup
2) Examine the potential influence of key confounding variables
3) Enhance the external validity of the conclusion for diverse patient population.

## 3 Results

### 3.1 Patients enrolled

We initially identified 1,405 hospitalized COVID-19 patients through electronic medical record review, we implement the following exclusion criteria: Six patients (0.4%) with negative COVID-19 nucleic acid tests, 709 (50.5%) lacking essential laboratory examinations, and 21 (1.5%) with follow-up durations of less than 3 days were excluded; 669 patients (47.6%) were included in the Azvudine-associated hepatotoxicity research (Figure 1). The enrolled population was subsequently stratified into two groups according to hepatotoxicity status, with comprehensive clinical characteristics presented in Table 1.

**TABLE 1:** Demographic characteristics of hospitalized COVID-19 patients in Azvudine-associated hepatotoxicity study.

Characteristics		Liver tests			Univariate analysis		Multivariate analysis	
		Total (n = 669, 100%)	Normal (n = 354, 52.9%)	Abnormal (n = 315, 47.1%)	OR (95% CI)	*P* value	OR (95% CI)	*P* value
Gender, n (%)	Male	446 (66.7)	218 (61.6)	228 (72.4)	1.63 (1.18–2.27)	0.003**	1.38 (0.94–2.02)	0.102
	Female	223 (33.3)	136 (38.4)	87 (27.6)				
Age, year, median (range)		70.0 (59.0–79.0)	69.5 (59.0–79.0)	70.0 (60.0–79.0)	1.00 (0.99–1.01)	0.589		
Smoke, n (%)	No	581 (86.8)	315 (89.0)	266 (84.4)				
	Yes	88 (13.2)	39 (11.0)	49 (15.6)	1.49 (0.95–2.35)	0.084	1.10 (0.58–2.10)	0.764
Drink, n (%)	No	613 (91.6)	329 (92.9)	284 (90.2)				
	Yes	56 (8.4)	25 (7.1)	31 (9.8)	1.44 (0.83–2.51)	0.197	1.20 (0.56–2.62)	0.640
Allergic, n (%)	No	623 (93.1)	327 (92.4)	296 (94.0)				
	Yes	46 (6.9)	27 (7.6)	19 (6.0)	0.78 (0.42–1.42)	0.417		
Severity of COVID-19, n (%)	Non-severe	352 (52.6)	200 (56.5)	152 (48.3)				
	Severe	317 (47.4)	154 (43.5)	163 (51.8)	1.39 (1.03–1.89)	0.033*	0.90 (0.63–1.28)	0.559
Infection indicators tests on admission	Normal	183 (27.4)	107 (30.8)	76 (24.1)				
	Abnormal	486 (72.6)	247 (69.2)	239 (75.9)	1.36 (0.97–1.92)	0.078	0.91 (0.61–1.37)	0.665
Liver tests on admission	Normal	332 (49.6)	245 (69.2)	87 (27.6)				
	Abnormal	337 (50.4)	109 (30.8)	228 (72.4)	5.89 (4.23–8.27)	<0.001***	5.55 (3.94–7.90)	<0.001***
Comorbidities, n (%)	Liver diseases	88 (13.2)	46 (13.0)	42 (13.3)	1.03 (0.66–1.61)	0.897		
	Renal diseases	208 (30.3)	108 (30.5)	100 (31.8)	1.06 (0.76–1.47)	0.730		
	Hypertension	248 (37.1)	135 (38.1)	113 (35.9)	0.91 (0.66–1.24)	0.545		
	Hyperlipidemia	18 (2.7)	8 (2.3)	10 (3.2)	1.42 (0.55–3.76)	0.468		
	Hypoproteinemia	400 (59.8)	209 (59.0)	191 (60.6)	1.07 (0.78–1.46)	0.674		
	Diabetes	123 (18.4)	68 (19.2)	55 (17.5)	0.89 (0.60–1.32)	0.560		
	Cancer	92 (13.8)	56 (15.8)	36 (11.4)	0.69 (0.43–1.07)	0.101	0.67 (0.38–1.15)	0.145
Azvudine therapy	Treatment duration, days, mean (±SD)	8.8 (±3.8)	8.9 (±3.6)	8.6 (±4.1)	0.98 (0.94–1.02)	0.286		
	Accumulated dose, mg, mean (±SD)	43.1 (±18.8)	44.0 (±17.2)	42.1 (±20.4)	1.00 (0.99–1.00)	0.200		
Concomitant medication, n (%)	Anti-viral drugs	73 (10.9)	31 (8.8)	42 (13.3)	1.60 (0.98–2.64)	0.060	1.35 (0.77–2.39)	0.303
	Anti-bacterial drugs	632 (94.5)	330 (93.2)	302 (95.9)	1.69 (0.86–3.47)	0.138	1.23 (0.56–2.77)	0.608
	Anti-fungal drugs	69 (10.3)	36 (10.2)	33 (10.5)	1.03 (0.63–1.70)	0.896		
	Respiratory drugs	485 (72.5)	258 (72.9)	227 (72.1)	0.96 (0.68–1.35)	0.813		
	Anti-hypertensive drugs	258 (38.6)	124 (35.0)	134 (42.5)	1.37 (1.01–1.88)	0.047*	1.16 (0.80–1.67)	0.426
	Stain	95 (14.2)	43 (12.1)	52 (16.5)	1.43 (0.93–2.22)	0.108	1.07 (0.64–1.80)	0.792
	Antiplatelet agents	80 (12.0)	42 (11.9)	38 (12.1)	1.02 (0.64–1.63)	0.937		
	Anticoagulant	338 (50.5)	150 (42.4)	188 (59.7)	2.01 (1.48–2.74)	<0.001***	1.79 (1.27–2.54)	0.001^**^
	Anti-diabetic drugs	137 (20.5)	68 (19.2)	69 (21.9)	1.18 (0.81–1.72)	0.389		
	Glucocorticoids	554 (82.8)	285 (80.5)	269 (85.4)	1.42 (0.95–2.14)	0.095	1.25 (0.79–2.00)	0.349
	NSAIDs	148 (22.1)	75 (21.2)	73 (23.2)	1.12 (0.78–1.62)	0.536		
	PPIs	493 (73.7)	258 (72.9)	235 (74.6)	1.09 (0.78–1.55)	0.614		
	Anti-neoplastic drugs	27 (4.0)	18 (5.1)	9 (2.9)	0.55 (0.23–1.21)	0.149	0.90 (0.34–2.26)	0.821

Abbreviations: COVID-19, Coronavirus disease 2019; OR, Odds ratio; CI, Confidence interval; SD, Standard deviation; NSAIDs, Nonsteroidal anti-inflammatory drugs; PPIs, Proton pump inhibitors.

Note: *means *P* < 0.05. **means *P* < 0.01. ***means *P* < 0.001.

To establish a baseline of normal liver function prior to treatment, we implement additional exclusion criteria: 88 (13.2%) cases were excluded due to liver disease, and 287 (42.9%) cases were excluded due to abnormal liver function, the remaining 294 (43.9%) cases enrolled in the study of Azvudine-induced hepatotoxicity (Figure 1). The final cohort was dichotomized based on the development of treatment-emergent liver injury, with comprehensive demographic and clinical characteristics detailed in Table 2.

**TABLE 2:** Demographic characteristics of hospitalized COVID-19 patients in Azvudine-induced hepatotoxicity study.

Characteristics		Liver tests			Univariate analysis		Multivariate analysis	
		Total (n = 294, 100%)	Normal (n = 214, 72.8%)	Abnormal (n = 80, 27.4%)	OR (95% CI)	*P* value	OR (95% CI)	*P* value
Gender, n (%)	Male	178 (60.5)	123 (57.5)	55 (68.8)	1.63 (0.95–2.84)	0.080	1.77 (0.994–3.25)	0.057
	Female	116 (39.5)	91 (42.5)	25 (31.3)				
Age, year, median (range)		69.0 (59.0–78.0)	68.0 (58.0–78.0)	70.0 (58.0–78.0)	1.00 (0.98–1.02)	0.933		
Smoke, n (%)	no	266 (90.5)	195 (91.1)	71 (88.8)				
	yes	28 (9.5)	19 (8.9)	9 (11.3)	1.30 (0.54–2.94)	0.538		
Drink, n (%)	no	276 (93.9)	203 (94.9)	73 (91.3)				
	yes	18 (6.1)	11 (5.1)	7 (8.8)	1.77 (0.63–4.67)	0.256		
Allergic, n (%)	no	272 (92.5)	197 (92.1)	75 (93.8)				
	yes	22 (7.5)	17 (7.9)	5 (6.3)	0.77 (0.25–2.03)	0.624		
Severity of COVID-19, n (%)	Non-severe	178 (60.5)	136 (63.6)	42 (52.5)				
	Severe	116 (39.5)	78 (36.5)	38 (47.5)	1.58 (0.94–2.66)	0.086	1.28 (0.72–2.25)	0.399
Infection indicators tests on admission	Normal	99 (33.7)	76 (35.5)	23 (28.8)				
	Abnormal	195 (66.3)	138 (64.5)	57 (71.3)	1.36 (0.79–2.42)	0.276		
Comorbidities, n (%)	Renal diseases	90 (30.6)	63 (29.4)	27 (33.8)	1.22 (0.70–2.10)	0.476		
	Hypertension	110 (37.4)	78 (36.5)	32 (40.0)	1.16 (0.68–1.96)	0.576		
	Hyperlipidemia	7 (2.4)	4 (1.9)	3 (3.8)	2.05 (0.40–9.48)	0.356		
	Hypoproteinemia	168 (57.1)	119 (55.6)	49 (61.3)	1.26 (0.75–2.15)	0.385		
	Diabetes	67 (22.8)	53 (24.8)	14 (17.5)	0.64 (0.33–1.21)	0.188	0.62 (0.29–1.24)	0.191
	Cancer	41 (14.0)	31 (15.0)	10 (12.5)	0.84 (0.38–1.76)	0.662		
Azvudine therapy	Treatment duration, days, mean (±SD)	8.9 (±3.5)	8.7 (±3.5)	9.5 (±3.4)	1.05 (0.98–1.13)	0.163	1.01 (0.93–1.09)	0.903
	Accumulated dose, mg, mean (±SD)	43.5 (±16.5)	42.7 (±16.1)	46.2 (±17.2)	1.01 (1.00–1.03)	0.209		
Concomitant medication, n (%)	Anti-viral drugs	22 (7.5)	10 (4.7)	12 (15.0)	3.60 (1.49–8.89)	0.004**	3.80 (1.47–10.1)	0.006**
	Anti-bacterial drugs	271 (92.5)	196 (91.6)	75 (93.8)	1.38 (0.53–4.29)	0.541		
	Anti-fungal drugs	31 (10.5)	19 (8.9)	12 (15.0)	1.81 (0.82–3.89)	0.132		
	Respiratory drugs	211 (71.8)	153 (71.5)	58 (72.5)	1.05 (0.60–1.89)	0.865		
	Anti-hypertensive drugs	100 (34.0)	67 (31.3)	33 (41.3)	1.54 (0.90–2.62)	0.111	1.37 (0.75–2.50)	0.305
	Stain	35 (11.9)	22 (10.3)	13 (16.3)	1.69 (0.79–3.51)	0.163	1.51 (0.66–3.41)	0.320
	Antiplatelet agents	32 (10.9)	23 (10.7)	9 (11.3)	1.05 (0.44–2.31)	0.902		
	Anticoagulant	132 (44.9)	79 (36.9)	53 (66.3)	3.35 (1.97–5.82)	<0.001***	3.12 (1.77–5.61)	<0.001***
	Anti-diabetic drugs	58 (19.7)	43 (20.1)	15 (18.8)	0.92 (0.47–1.73)	0.797		
	Glucocorticoids	236 (80.3)	169 (79.0)	67 (83.8)	1.37 (0.71–2.80)	0.361		
	NSAIDs	61 (19.6)	43 (20.1)	18 (22.5)	1.15 (0.61–2.13)	0.651		
	PPIs	223 (75.9)	161 (75.2)	62 (77.5)	1.13 (0.63–2.13)	0.686		
	Anti-neoplastic drugs	14 (4.8)	12 (5.6)	2 (2.5)	0.43 (0.07–1.63)	0.278		

Abbreviations: COVID-19, Coronavirus disease 2019; OR, Odds ratio; CI, Confidence interval; SD, Standard deviation; NSAIDs, Nonsteroidal anti-inflammatory drugs; PPIs, Proton pump inhibitors.

Note: *means *P* < 0.05. **means *P* < 0.01. ***means *P* < 0.001.

### 3.2 Characteristics of COVID-19 patients with Azvudine-associated hepatotoxicity

Our analysis of 669 hospitalized COVID-19 patients revealed that 315 cases (47.1%) exhibited abnormal liver function test, whereas 354 patients (52.9%) maintained normal hepatic parameters, as shown in Table 1. Multivariate logistic regression identified two significant risk factors for hepatoxicity. (1) male gender (OR 1.63, 95% CI 1.18–2.27, *P =* 0.003), and (2) severe COVID-19 infection (OR 1.39, 95% CI 1.03–1.89, *P =* 0.033) The conclusion suggest that male patients and those with severe COVID-19 manifestations demonstrate increased susceptibility to Azvudine-associated injury.

**FIGURE 2:**
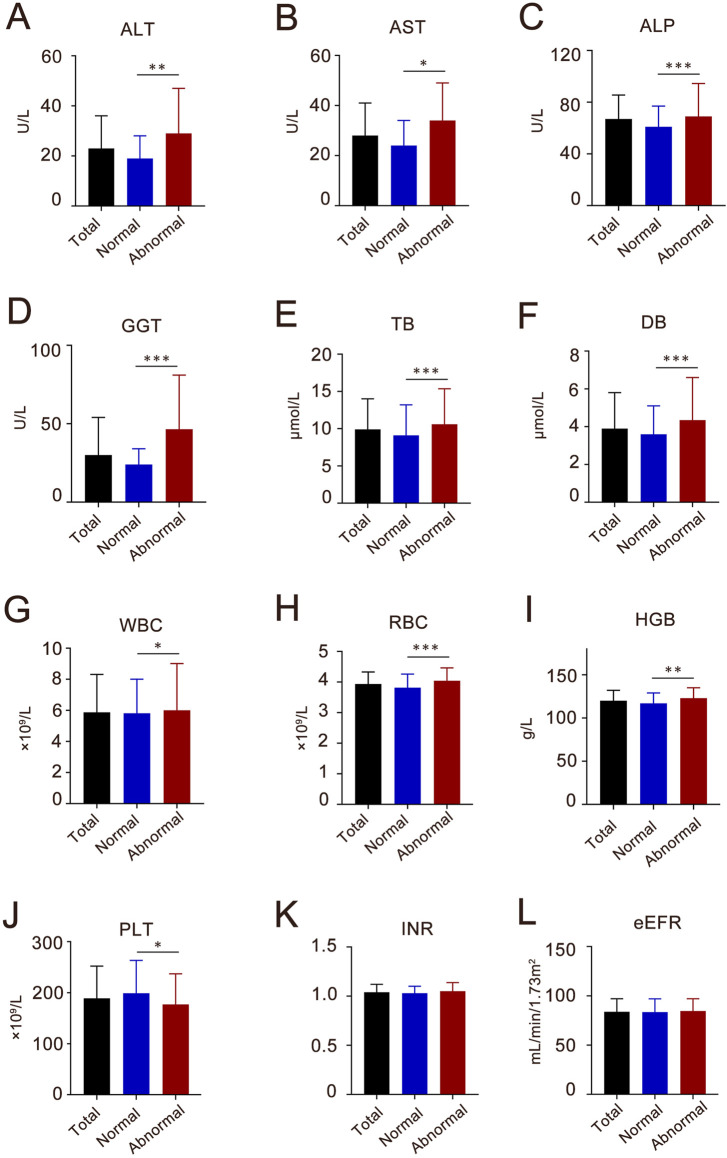
The baseline characteristics of liver enzymes, blood components, coagulate and kidney function index of the participants in Azvudine-associated hepatotoxicity study. **(A)** ALT, alanine aminotransferase; **(B)** AST, aspartate transaminase; **(C)** ALP, alkaline phosphatase; **(D)** GGT, gamma–glutamyltransferase; **(E)** TB, total bilirubin; **(F)** DB, direct bilirubin; **(G)** WBC, white blood cell; **(H)** RBC, red blood cell; **(I)** HGB, hemoglobin; **(J)** PLT, platelet count; **(K)** INR, international normalized ratio; **(L)** eGFR, estimated glomerular filtration rate. Each column shows the median (interquartile range, IQR). *P < 0.05, **P < 0.01, ***P < 0.001.

The analysis identified several significant predictors of Azvudine associated hepatotoxicity Notably, abnormal liver function tests at admission demonstrated the strongest association (OR 5.89, 95% CI 4.28–8.27, *P* < 0.001). Interestingly, the presence of comorbidities did not significantly affect liver function disorders in hospitalized patients. However, the concurrent use of antihypertensive (OR1.37, 95% CI 1.01–1.88, *P =* 0.047) and anti-thrombotic drugs (OR 2.01, 95% CI 1.48–2.74, *P <* 0.001) was significantly correlated with abnormal liver function. Comparative analysis of baseline laboratory parameters (Figure 2) revealed significant differences (*P* < 0.05) in multiple hepatic markers (ALT, AST, ALP, GGT, TB, and DB), and hematological indices (WBC, RBC, HGB, and PLT) between groups. Notably, coagulation such as the international normalized ratio (INR) and renal function parameters such as estimated glomerular filtration rate (eGFR) showed no significant difference intergroup variation.

The multivariate logistic regression analysis incorporating demographic characteristics (gender, smoking, drinking), clinical factors (severity of COVID-19), admission laboratory tests (infection indicator and liver function), and medication history (antiviral drugs, antibacterial drugs, antihypertensive drugs, statins, anticoagulants, glucocorticoids, and antineoplastic drugs) on adimission were subsequently incorporated to further identified their associations. (1) Abnormal liver function tests on admission (OR 5.55, 95% CI 3.94–7.90, *P <* 0.001). (2)Antithrombotic medication management (OR 1.79, 95% CI 1.27–2.54, *P =* 0.001). The model demonstrated good discriminatory ability with an area under the ROC curve of 0.756, 95% CI 0.719–0.792, *P <* 0.001), indicating moderate predictive accuracy for liver injury risk in COVID-19 patients receiving Azudine.

### 3.3 Characteristics of COVID-19 patients with Azvudine-induced hepatotoxicity

**FIGURE 3:**
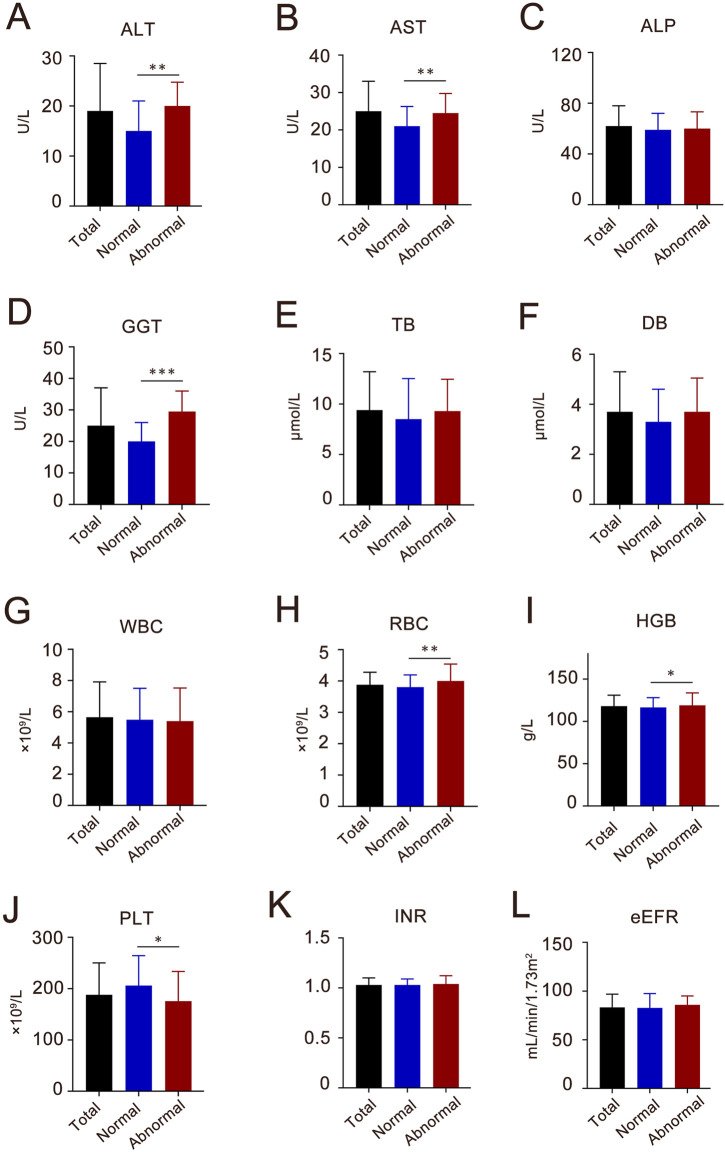
The baseline characteristics of liver enzymes, blood components, coagulative and kidney function index of the participants in Azvudine-induced hepatotoxicity study. **(A)** ALT, alanine aminotransferase; **(B)** AST, aspartate transaminase; **(C)** ALP, alkaline phosphatase; **(D)** GGT, gamma–glutamyltransferase; **(E)** TB, total bilirubin; **(F)** DB, direct bilirubin; **(G)** WBC, white blood cell; **(H)** RBC, red blood cell; **(I)** HGB, hemoglobin; **(J)** PLT, platelet count; **(K)** INR, international normalized ratio; **(L)** eGFR, estimated glomerular filtration rate. Each column shows the median (interquartile range, IQR). *P < 0.05, **P < 0.01, ***P < 0.001.

Overall, 80 (27.2%) patients developed hepatoxicity after Azvudine treatment, while 214 (72.8%) maintained normal. Multivariate analysis identified two independent predictors: concomitant antiviral therapy (adjusted OR 3.60, 95%CI 1.49–8.89, *P* = 0.004); Antithrombotic medication management (adjusted OR 3.35, 95% CI 1.77–5.61, *P* < 0.010). Additionally, the regression model demonstrated acceptable discrimination (AUC 0.712, 95% CI 0.648–0.775, *P* < 0.001). For laboratory examination, significant post-treatment elevations in hepatic enzymes (baseline levels of ALT, AST, GGT, WBC, HGB, and PLT) were significantly associated with liver injury (all *P <* 0.05, Figure 3). Gender, COVID-19 severity, diabetes, Azvudine therapy, antiviral drugs, and antithrombotic drugs were then incorporated into a multivariate LR model. It was determined that anti-viral (OR 3.80, 95% CI 1.47–10.1, *P =* 0.006) and Anti-thrombotic drugs (OR 3.12, 95%CI 1.77–5.61, *P <* 0.001) were both indendent predictors of Azudine hepatotoxicity. The sensitivity analysis results were consistent with the primary analysis, thus lending support of the accuracy of the conclusion (Supplementary Table S1). Additionally, a significant elevation in liver function (ALT, AST, and GGT) was revealed following Azvudine administration (Table 3), indicating the occurrence of liver injury (*P <* 0.05).

**TABLE 3:** The liver tests result of Azvudine on and after admission among hospitalized COVID-19 patients with Azvudine-induced hepatotoxicity.

Liver tests		Azvudine treatment		*P* value
		On admission	After admission	
ALT (U/L), median (range)	Total	23.0 (14.0–36.0)	26.00 (15.00–49.00)	<0.001***
	Normal	19.0 (12.0–28.0)	18.00 (12.00–25.3)	0.182
	Abnormal	29.0 (19.0–47.0)	52.0 (30.0–79.0)	<0.001***
AST (U/L), median (range)	Total	28.0 (21.0–41.0)	22.00 (16.00–36.00)	0.038*
	Normal	24.0 (18.0–34.0)	17.5 (14.00–22.00)	<0.001***
	Abnormal	34.0 (24.0–49.0)	37.0 (24.0–58.0)	<0.001***
ALP(U/L), median (range)	Total	67.0 (54.5–85.5)	66.00 (54.00–85.00)	0.160
	Normal	61.0 (53.0–77.0)	59.00 (50.00–71.5)	0.918
	Abnormal	69.0 (57.0–94.5)	74.0 (59.0–100.0)	0.025*
GGT (U/L), median (range)	Total	30.0 (20.0–54.0)	34.00 (22.00–63.25)	<0.001***
	Normal	24.0 (17.0–34.0)	23.00 (18.00–31.00)	0.080
	Abnormal	46.5 (27.8–81.0)	74.0 (59.0–100.0)	<0.001***
TB (µmol/L), median (range)	Total	9.9 (7.1–14.0)	9.70 (7.40–13.20)	0.633
	Normal	9.1 (6.9–13.2)	8.6 (6.9–12.0)	0.344
	Abnormal	10.6 (7.7–15.4)	10.7 (7.8–14.8)	0.594
DB (µmol/L), median (range)	Total	3.9 (2.8–5.8)	3.60 (2.60–5.20)	0.410
	Normal	3.6 (2.5–5.1)	3.2 (2.3–4.4)	0.384
	Abnormal	4.4 (3.1–6.6)	4.1 (2.9–6.3)	0.826

Abbreviations: COVID-19, Coronavirus disease 2019; ALT, alanine aminotransferase; AST, aspartate transaminase; ALP, alkaline phosphatase; GGT, gamma–glutamyltransferase; TB, total bilirubin; DB, direct bilirubin.

Note: *means *P* < 0.05. **means *P* < 0.01. ***means *P* < 0.001.

### 3.4 Concomitant medication of Azvudine-induced hepatotoxicity in COVID-19 patients

To identify concomitant medications significantly associated with Azvudine-induced hepatotoxicity, we conducted focused analysis of pharmacological agents that demonstrated statistical significance (*P* < 0.05) in our multivariate regression model, as shown in Table 4. Significant Hepatotoxicity Associations: Ganciclovir (adjusted OR 4.22, 95% CI 1.56–12.0, *P* = 0.005). LMWH showed significant risk (OR 3.01, 95% CI 1.73–5.23, *P* < 0.001). Multivariate Analysis Results: The final predictive model identified three independent pharmacological risk factors: Ganciclovir (adjusted OR 4.11, 95% CI 1.45–12.2, *P* = 0.008). LMWH calcium (adjusted OR 3.00, 95% CI 1.69–5.33, *P* < 0.001). Enoxaparin (adjusted OR 2.68, 95% CI 0.99–7.10, *P* = 0.047). Model Performance: The model achieved acceptable discrimination (AUC 0.674, 95% CI 0.602–0.746, *P* < 0.001). Validation: Robustness confirmed through consistent sensitivity analyses (Supplementary Table S2). All significant associated maintained statistical significance in validation testing.

**TABLE 4:** Concomitant medication of hospitalized COVID-19 patients in Azvudine-induced hepatotoxicity study.

Concomitant medication		Liver tests			Univariate analysis		Multivariate analysis	
		Total (n = 294, 100%)	Normal (n = 214, 72.8%)	Abnormal (n = 80, 27.4%)	OR (95% CI)	*P* value	OR (95% CI)	*P* value
Anti-viral drugs	Ganciclovir	17 (5.8)	7 (3.3)	10 (12.5)	4.22 (1.56–12.0)	0.005**	4.11 (1.45–12.2)	0.008^**^
		5 (1.7)	3 (1.4)	2 (2.5)	1.80 (0.234–11.1)	0.523		
Anticoagulants	Low Molecular Weight Heparin Calcium	79 (26.9)	44 (20.6)	35 (43.8)	3.01 (1.73–5.23)	<0.001***	3.00 (1.69–5.33)	<0.001***
	Low Molecular Weight Heparin Sodium	8 (2.7)	4 (1.9)	4 (5.0)	2.76 (0.64–11.9)	0.158	2.97 (0.65–13.5)	0.146
	Enoxaparine	20 (6.8)	11 (5.1)	9 (11.3)	2.34 (0.91–5.88)	0.071	2.68 (0.99–7.10)	0.047^*^
	Nadroparin	23 (7.8)	17 (7.9)	6 (7.5)	0.94 (0.33–2.36)	0.900		
	NOACs	15 (5.1)	12 (5.6)	3 (3.8)	0.66 (0.15–2.13)	0.522		
	Sulodexide	27 (9.2)	23 (10.7)	4 (5.0)	0.44 (0.13–1.18)	0.138	0.53 (0.15–1.49)	0.269

Abbreviations: COVID-19, Coronavirus disease 2019; OR, Odds ratio; CI, Confidence interval; NOACs, new oral anticoagulants.

Note: *means *P* < 0.05. **means *P* < 0.01. ***means *P* < 0.001.

### 3.5 Clinical outcome of Azvudine-induced hepatotoxicity, severity, and treatment

The characteristics and outcomes of the Azvudine-induced hepatotoxicity was shown in Table 5. Severity Distribution: Grade 1 (Mild): 64 cases (80.0%); Grade 2 (Moderate): 14 cases (17.5%); Grade 3 (Grade 3+): 2 cases (2.5%). The onset of liver injury occurred at approximately 7 days (range 5–10 days) and showed no significant differences across severity grades.

**TABLE 5:** Clinical characteristics and outcome of hospitalized COVID-19 patients in Azvudine-induced hepatotoxicity study.

Characteristics		Liver tests				
		Total (n = 80, 100%)	Grade 1 (n = 64, 80.0%)	Grade 2 (n = 14, 17.5%)	Grade 3+ (n = 2, 2.5%)	*P* value
Time of onset, median (range)		7.0 (5.0–10.0)	6.0 (5.0–10.0)	9.0 (6.8–11.3)	7.5 (5.0–10.0)	0.2204
RUCAM score	3–5	41 (51.3)	34 (53.1)	6 (42.9)	1 (50.0)	0.880
	6–8	37 (46.3)	28 (43.8)	8 (57.1)	1 (50.0)	
	>8	2 (2.5)	2 (3.1)	0(0.0)	0 (0.0)	
Clinical classification	Hepatocyte type	16 (20.0)	8 (12.5)	6 (42.9)	2 (100.0)	0.005**
	Cholestasis type	18 (22.5)	15 (23.4)	3 (21.4)	0 (0.0)	
	Mixed type	46 (57.5)	41 (64.1)	5 (35.7)	0 (0.0)	
Hepatoprotective drugs	Glycyrrhizin drugs	15 (18.8)	12 (18.8)	1 (7.1)	2 (100.0)	0.703
	Glutathione	19 (23.8)	15 (23.4)	3 (24.4)	1 (50.0)	
	Polyene phosphatidyl choline	6 (7.5)	4 (6.3)	2 (14.3)	0 (0.0)	
	Other drugs	6 (7.5)	4 (6.3)	1 (7.2)	1 (50.0)	
Clinical outcome	Recovery	33 (41.3)	29 (45.3)	4 (28.6)	0 (0.0)	0.002**
	Improvement	37 (46.3)	28 (43.8)	9 (64.3)	1 (50.0)	
	Progression	3 (3.8	1 (1.6)	0 (0.0)	1 (50.0)	
	Unknown	7 (8.8)	6 (9.4)	1 (7.2)	0 (0.0)	
All-cause mortality		5 (7.5)	2 (3.1)	1 (7.2)	2 (100.0)	<0.001***

Abbreviations: COVID-19, Coronavirus disease 2019; RUCAM, Roussel Uclaf Causality Assessment Method.

Note: **means *P* < 0.01. ***means *P* < 0.001.

Causality and Phenotypic Characterization of Azvudline-Induced Hepatotoxicity. RUCAM-based Causality Assessment: Possible association (score 3–5): 41 cases (51.3%); Probable association (score 6–8): 37 cases (46.2%). Highly probable association (score>8): 2 cases (2.5%). Injury Patterns at Presentation: Hepatocellular (R ≥ 5): 16 cases (20.0%); Cholestatic (R ≤ 2): 18 cases (22.5%). Mixed (2 < R < 5):46 cases (57.5%).

There was a significant difference between the level of liver injury: mild cases predominantly showed mixed pattern, while severe cases demonstrated hepatocellular predominance. Notably, two cases in the hepatocellular pattern reached grade 3 and one case achieved grade 4, with an increasing AST value of 585 U/L (>10X the upper limit of normal, ULN).

As for treatment patterns: 32/80 (40.0%) patients received hepatoprotective therapy. Medication utilization:Glutathione (19, 23.75%) was the most frequently administered treatment for DILI, followed by Glycyrrhizin (15, 18.75%) and Polyene phosphatidylcholine (6, 7.5%). Treatment Outcome: The majority of patients achieved recovery or improvement after therapy, although two patients showed disease progression despite hepatoprotection. Case presentations of progressive liver injury: One patient initially presented with mild liver injury (Grade 1) with cholestatic pattern that progressed to acute liver failure within 1 week, exhibiting terminal hepatocellular pattern changes with AST levels of 684 U/L, ALT >6000 U/L, ALP 161 U/L, and GGT 79 U/L, whereas TB and DB levels remained normal at 8.3 μmol/L and 5.3 μmol/L, respectively. Another patient persistent AST level of >200 U/L with severe liver injury maintained and fatal outcome due to severe pneumonia complications 4 days post discontinuation of Azvudine. Clinical Observation: Although progress cases received hepatoprotective therapy, rapid biochemical deterioration occurred despite standard interventions. Non-hepatic comorbidties contributed to mortality in severe cases.

Mortality analysis in the study cohort. Overall Mortality in entire cohort, 14 (4.76%, 14/294) died after Azvudine therapy. In the normal liver function group, the all-cause mortality rate was 4.21% (9/214), with five patients dying from severe pneumonia, one from MODS, and one from acute heart failure (AHF). The all-cause mortality by hepatotoxicity severity in the liver injury group was 6.25% (5/80), with 2 cases (3.13%, 2/64) Grade 1, 1 case (7.14%, 1/14) Grade 2, and 2 cases (100%, 2/2) Grade 3 or higher. Among which, four patients died from severe pneumonia and one died from MODS. Although there was no significant difference between the normal and abnormal liver function groups, severe liver injury significantly contributed to the progression of the main disease. Liver injury severity correlated with worse clinical outcomes, particularly in patients with pre-existing severe COVID-19. Hepatotoxicity may potentiate disease progression in critical ill patients.

## 4 Discussion

During the COVID-19 pandemic, the urgent need for the effective antiviral medication has highlighted the importance of rigorous drug safety evaluation. Azvudine has emerged as a promising newly marketed medication, its safety profile, particularly regarding hepatotoxicity remains incompletely characterized, especially for off-label use in hospitalized patients with prolonged symptomatic combined with complex commodities. In the initial studies, the medication did not demonstrate any significant adverse effects, indicating a seemingly safe profile for its application. Nevertheless, this is the first large-scale evaluation of Azvudine hepatotoxicity in real-world hospitalized patients. The research focus on high-risk population with symptoms prolonged more than 5 days with multiple comorbidities/concomitant medications. Sample size (n = 1405) substantially exceeds previous clinical trial cohorts.

Previous studies have documented that hepatocellular injury in 14%–53% of hospitalized COVID-19 patients, typically characterized by an elevation in aminotransferase levels below 5-fold ULN in initial stage of pandemic (Gupta et al., 2020). In our prior single center data demonstrated progress from 51.2% admission prevalence to 70.0% during hospitalization (Chen et al., 2023). Recently, multicenter study enrolling 1246 hospitalized adult patients identified that approximately 58.7% patients had presented with abnormal liver biochemistry and 47.7% had persistent abnormalities up to 6 months post-infection (Rajaram et al., 2024). In our study, the overall hepatoxicity prevalence: 47.1% consistent with history ranges. Characteristic pattern: isolated with elevated levels of ALT, AST, and GGT, indicating that Azvudine did not increase the risk of hepatotoxicity compared with other anti-viral therapies in COVID-19 treatment.

In our cohort, the incidence of Azvudine-induced hepatotoxicity was 27.2%. Most of cases were characterized by mild-to-moderate hepatic impairment, corresponding to Grades 1 and 2. In previous studies, varying incidences of hepatic impairment have been documented among COVID-19 patients of diverse types when administered Azvudine therapy, with reported rates of liver injury ranging from 10.5% (Lan et al., 2024) to 17.8% (Yang et al., 2023), 18.6% (Liu S. et al., 2023), 23.0% (Zhi et al., 2023) and 38.4% (He et al., 2023). The retrospective study conducted by Liu et al., among 490 patients with mild COVID-19 who underwent pharmacological treatment, 91 individuals presented with aberrant hepatic function, predominantly manifested as elevated liver enzyme levels (Liu ZQ. et al., 2023). The meticulous study conducted by Li et al., with cohort of 190 patients who received Azvudine demonstrated an incidence of elevated ALT of 38.4% (73/190) (He et al., 2023). The majority of these cases, specifically 93.2% (68/73) patients exhibited an increase within one-fold the ULN and zero case up to 5-fold, which is indicative of a benign pattern typically observed in clinical trials. Age specific hepatotoxicity profile of Azvudine therapy. Elderly patients aged over 75 years old with the rate of abnormal liver function of 23.0% (17/74), among which 6 occurrences of elevated ALT, 4 of elevated AST, and 7 of elevated GGT (Zhi et al., 2023). Notably, the incidence of Azvudine-induced DILI was observed to be relatively as low as 0.68% (2/294) in our study. Consistent findings were reported in another cohort by Zhou et al., with Azvudine-induced DILI of 0.64% (2/311) in the treatment for moderate to severe COVID-19 patients (Zhou et al., 2023). Liu et al. conducted the study involving 294 patients, of which 17 cases of DILI were identified (Liu et al., 2024). The higher incidence may be attributed to the inclusion of patients with pre-existing elevated liver function at the enrollment stage, which could not only increase the risk of occurrence but also confounds non-pharmacological factors. In summary, the aggregate data from these studies suggest that Azvudine was associated with a favorable safety profile in the context of COVID-19 therapy.

Medications have been identified as one of the causes of hepatocellular damage in COVID-19 infections (Zhong et al., 2020; Wen-Shu Hu, 2022; Rodriguez-Espada et al., 2024). In previous researches, antiviral drugs, Remdesivir, and Nirmatrelvir-Ritonavir have been reported to have greater risk of developing drug-induce hepatotoxicity (Allison et al., 2023; Xie et al., 2020; Naseralallah et al., 2022). During the Azvudine therapy in our cohort, the value of liver enzymes increased significantly, revealing the potential hepatotoxicity. However, the mechanism underlying Azvudine-induced hepatic injury in COVID-19 patients remained unclear. Naveen et al. have demonstrated that Azvudine could cause mitochondrial ROS induction by two functional groups on sugar, Azido at 4′-poisoning and fluorine at 2′-poisoning, induce ROS generation with a time- and dose-dependent manner, prompt mitochondrial-mediated apoptosis (Kumar et al., 2024b; Kumar et al., 2024a). However, due to its minimal plasma protein binding affinity, Azvudine exhibited a propensity for accumulation in the thymus and was excreted in its parent form via renal clearance, which seems to be a safety pattern in the liver system (He et al., 2023; Liu S. et al., 2023; Sheng et al., 2024). Based on current pharmacokinetic and toxicity data, we assumed that the administration of Azvudine might lead to a dose-dependent intrinsic hepatotoxicity due to the accumulation exceeding the threshold level as a consequence of impaired excretion.

Interestingly, in the present study only concomitant antiviral and anticoagulant drugs were independent risk factors for Azvudine-induced hepatotoxicity, whereas other factors, including gender, age, severity of COVID-19, and comorbidities, were not significantly associated with the risk of liver injury. Further examination revealed that the co-administration of Ganciclovir, Low-Molecular-Weight Heparin Calcium, and Enoxaparin increased the risk. Literatures have indicated an increased likelihood of adverse drug reactions with the concurrent use of multiple antiviral agents (Liu et al., 2024). This might be attributed to competitive targeting by these drugs, resulting in cellular damage (Abdallah et al., 2014). Therefore, the concomitant use of multiple antiviral agents in clinical settings was not advocated. Additionally, it is suggested the concurrent use of anticoagulant drugs contributed not only the Azvudine but also Azvudine-induced hepatotoxicity. These conclusions aligned with previous studies, which identified anticoagulant as an independent risk factor for liver injury and increased 28-day mortality in COVID-19 patients, underscoring the potential correlation that warrants further investigation (Chen et al., 2024; Lu et al., 2024). Retrospective cohort study conducted by Yang et al., anticoagulants (LMWH and Fondaparinux) were associated with liver dysfunction with occurrence rate of 17.1% (79/463) in pulmonary embolism patients, recommencing transit to oral anticoagulants if possible (Yang et al., 2020). These findings were also recommended in our research as the new oral anticoagulants (NOACs) showed no significant difference in hepatotoxicity. Elder age and hypoproteinemia have been identified as risk factors for the progress of Azvudine-induced hepatotoxicity in previous studies (Lan et al., 2024; Zhi et al., 2023; Lu et al., 2024). However, the factors were not obvious in our study, possibly due to the inclusion of patients with a median age of >65 years, and the prevalence of critically ill patients contributed to lower baseline albumin level, which could have obscured the observed differences in the analysis.

In this study, the relatively low RUCAM scores, with only two cases indicating a highly probable association, suggested a tenuous causal relationship between Azvudine and hepatotoxicity (Danan and Teschke, 2018; Caines and Moonka, 2024; Zhao et al., 2024). This observation likely stems from multiple factors. Notably, polypharmacy was prevalent, the concurrent use of hepatotoxic agents with established hepatotoxic potential, such as statins and NSAIDs, complicated the assessment of Azvudine’s specific contribution to hepatotoxicity (Teschke et al., 2022; Russo et al., 2014; Zoubek et al., 2020). Additionally, the absence of rechallenge data posed a significant challenge. Most patients did not reintroduce the suspected medication post-discontinuation, creating an evidentiary gap in a critical RUCAM evaluation component. This lack of rechallenge documentation made definitively establishing a drug-hepatotoxicity link particularly difficult. Furthermore, the study’s relatively small positive sample size may have contributed to the lower RUCAM scores. Future research will address these limitations through large-scale studies and more comprehensive hepatotoxicity assessments, with the goal of clarifying the causal relationship between Azvudine and hepatotoxicity.

Although our study has several limitations, the conclusion provide great significance insights into hepatotoxicity associated with antiviral therapy in COVID-19 patients. First, as restrospective analysis, potential biases may existed due to incomplete or inconsistent data recording in electronic medical records (e.g., Incomplete body mass index). Second, the available data on combining medications were insufficient for robust statistical analysis, and the result was still suspicious and required further validation in future studies. Additionlly, the lack of standardized follow up protocols limited our ability to monitor liver injury progression and management during Azvudine therapy in real time. To address these limitations, multicenter prospective study with larger sample sizes to comprehensively assess the real-world hepatoxicity profile of Azudine. The future research will incorporate structed follow-up schedules, standardized laboratory monitoring, and detailed documentation of concomitant medications to enhance data reliability and clinical applicability.

## 5 Conclusion

Our investigation demonstrated a significant association between Azvudine and hepatotoxicity, with a notably elevated incidence of 27.2%. Most of these hepatotoxic events were characterized by mild severity and were reversible upon drug withdrawal. Thus, it was essential for clinicians to monitor the hepatic function vigilantly throughout the therapeutic course. Moreover, the incidence of Azvudine-induced hepatotoxicity appeared to be exacerbated by the concomitant administration of antiviral and anticoagulant therapies, specifically Ganciclovir, Low-molecular-weight heparin calcium, and Enoxaparin. Therefore, we do not recommend the concurrent use of Ganciclovir with Azvudine, and instead we suggest considering NOACs as a preferable alternative to LMWH for anticoagulation therapy in patients receiving Azvudine therapy.

## Data Availability

The raw data supporting the conclusions of this article will be made available by the authors, without undue reservation.
