# Supplementary material for: Real-world data of Azvudine-induced hepatotoxicity among hospitalized COVID-19 patients in China: a retrospective case-control study
Source: Front Pharmacol. 2025 Jun 4;16:1558054. doi: 10.3389/fphar.2025.1558054 (PMC12175845; doi:10.3389/fphar.2025.1558054)
Supplement: Supplementary file 1 [file DataSheet1.docx]

Supplementary material table 1 The Multivariate analysis result of patients characteristics in sensitivity analysis

| Characteristics | | Unadjusted | | Adjusted for age over 35 years | | Adjusted for non-allergic patients | |
| --- | --- | --- | --- | --- | --- | --- | --- |
|  |  | OR (95% CI) | *P* value | OR (95% CI) | *P* value | OR (95% CI) | *P* value |
| Gender | Male | 1.77(0.99-3.25) | 0.057 | 1.77(0.98-3.24) | 0.061 | 1.53(0.84-2.85) | 0.168 |
| Severity of COVID-19 | Severe | 1.28(0.72-2.25) | 0.399 | 1.24(0.70-2.20) | 0.454 | 1.33(0.74-2.38) | 0.338 |
| Comorbidities | Diabetes | 0.62(0.29-1.24) | 0.191 | 0.61(0.29-1.22) | 0.176 | 0.55(0.25-1.17) | 0.123 |
| Azvudine therapy | Treatment duration, days, mean (±SD) | 1.01(0.93-1.09) | 0.903 | 1.01(0.93-1.09) | 0.830 | 1.01(0.93-1.10) | 0.768 |
| Concomitant medication | Anti-viral drugs | 3.80(1.47-10.1) | 0.006^＊＊^ | 3.80(1.47-10.02) | 0.006^＊＊^ | 3.00(1.11-8.18) | 0.030^＊^ |
|  | Anti-hypertensive drugs | 1.37(0.75-2.50) | 0.305 | 1.53(0.81-2.86) | 0.185 | 1.37(0.70-2.63) | 0.348 |
|  | Stain | 1.51(0.66-3.41) | 0.320 | 1.41(0.60-3.21) | 0.417 | 1.48(0.61-3.49) | 0.377 |
|  | Anticoagulant | 3.12(1.77-5.61) | ＜0.001^＊＊＊^ | 3.04(1.72-5.48) | ＜0.001^＊＊＊^ | 3.05(1.70-5.56) | ＜0.001^＊＊＊^ |

Abbreviations: COVID-19, Coronavirus disease 2019. OR, Odds ratio. CI,Confidence interval. SD, Standard deviation.

Note: *means *P* < 0.05. **means *P* < 0.01. ***means *P* < 0.001.

Supplementary material table 2 The Multivariate analysis result of concomitant medication in sensitivity analysis

| Concomitant medication | | Multivariate analysis | | Adjusted for age over 35 years | | Adjusted for non-allergic patients | |
| --- | --- | --- | --- | --- | --- | --- | --- |
|  |  | OR (95% CI) | *P* value | OR (95% CI) | *P* value | OR (95% CI) | *P* value |
| Anti-viral drugs | Ganciclovir | 4.11(1.45-12.2) | 0.008^＊＊^ | 4.06(1.44-12.02) | 0.009^＊^ | 3.05(1.01-9.44) | 0.047^＊^ |
| Anticoagulants | Low Molecular Weight Heparin Calcium | 3.00(1.69-5.33) | <0.001^＊＊＊^ | 2.92(1.65-5.19) | ＜0.001^＊＊＊^ | 2.93(1.62-5.32) | ＜0.001^＊＊＊^ |
|  | Low Molecular Weight Heparin Sodium | 2.97(0.65-13.5) | 0.146 | 3.76(0.77-20.39) | 0.100 | 2.80(0.61-12.72) | 0.169 |
|  | Enoxaparine | 2.68(0.99-7.10) | 0.047^＊^ | 2.64(0.98-6.99) | 0.049^＊^ | 2.90(1.00-8.31) | 0.045^＊^ |
|  | Sulodexide | 0.53(0.15-1.49) | 0.269 | 0.54(0.15-1.53) | 0.288 | 0.50(0.14-1.41) | 0.233 |

Abbreviations: COVID-19, Coronavirus disease 2019. OR, Odds ratio. CI,Confidence interval.

Note: *means *P* < 0.05. **means *P* < 0.01. ***means *P* < 0.001.
